# Cytotoxic Plant Extracts towards Insect Cells: Bioactivity and Nanoencapsulation Studies for Application as Biopesticides

**DOI:** 10.3390/molecules25245855

**Published:** 2020-12-11

**Authors:** Ana I. F. Lopes, Mariana Monteiro, Ana R. L. Araújo, Ana Rita O. Rodrigues, Elisabete M. S. Castanheira, David M. Pereira, Pedro Olim, A. Gil Fortes, M. Sameiro T. Gonçalves

**Affiliations:** 1 Centre of Chemistry, Department of Chemistry, Campus of Gualtar, University of Minho, 4710-057 Braga, Portugal; pg35027@alunos.uminho.pt (A.I.F.L.); pg31434@alunos.uminho.pt (M.M.); rita_leite3@hotmail.com (A.R.L.A.); gilf@quimica.uminho.pt (A.G.F.); 2 Centre of Physics, Department of Physics, Campus of Gualtar, University of Minho, 4710-057 Braga, Portugal; ritarodrigues@fisica.uminho.pt (A.R.O.R.); ecoutinho@fisica.uminho.pt (E.M.S.C.); 3 REQUIMTE/LAQV, Laboratory of Pharmacognosy, Department of Chemistry, Faculty of Pharmacy, University of Porto, R. Jorge Viterbo Ferreira, 228, 4050-313 Porto, Portugal; dpereira@ff.up.pt (D.M.P.); up201706671@ff.up.pt (P.O.)

**Keywords:** biopesticides, bioinsecticides, *Ruta graveolens*, chlorpyrifos, nanoencapsulation

## Abstract

The potential of plant extracts as bioinsecticides has been described as a promising field of agricultural development. In this work, the extracts of *Punica granatum* (pomegranate), *Phytolacca americana* (American pokeweed), *Glandora prostrata* (shrubby gromwell), *Ulex europaeus* (gorce), *Tagetes patula* (French marigold), *Camellia japonica red* (camellia), *Ruta graveolens* (rue or herb-of-grace) were obtained, purified, and their activity against *Spodoptera frugiperda* (*Sf9*) insect cells was investigated. From the pool of over twenty extracts obtained, comprising different polarities and vegetable materials, less polar samples were shown to be more toxic towards the insect cell line *Sf*9. Among these, a dichloromethane extract of *R. graveolens* was capable of causing a loss of viability of over 50%, exceeding the effect of the commercial insecticide chlorpyrifos. This extract elicited chromatin condensation and the fragmentation in treated cells. Nanoencapsulation assays of the cytotoxic plant extracts in soybean liposomes and chitosan nanostructures were carried out. The nanosystems exhibited sizes lower or around 200 nm, low polydispersity, and generally high encapsulation efficiencies. Release assays showed that chitosan nanoemulsions provide a fast and total extract release, while liposome-based systems are suitable for a more delayed release. These results represent a proof-of-concept for the future development of bioinsecticide nanoformulations based on the cytotoxic plant extracts.

## 1. Introduction

Many synthetic chemicals have been used in plant protection against agroindustrial and medicinal pests for the last 60 years [1,2,3]. This has caused a strong negative impact at different levels, and there are serious concerns about their side effects, such as the presence of residues on food and drinking water, toxicity to humans as well as to other non-target organisms, and high persistence, leading to an emergence of pest resistance. These facts have demanded for a drastic decrease in the use of chemical pesticides and opening up space for new and safer alternative strategies [1]. The use of natural compounds to control weeds has long been accepted as an environment friendly strategy, but farmers have mostly relied on toxic synthetic agrochemicals. New tools in pest management are based on biopesticides, which include semiochemicals and plant-incorporated protectants, botanical and microbial derived chemicals, and also new synthetic analogues [1]. The major problems in organic agriculture faced by farmers are weed management, crop diseases and insect controlling tools. Due to the diversity of chemicals present, natural products have become important sources for most biological activity optimization, also playing also an important role in drug discovery [4]. In recent years, research has been focused on the pesticidal efficiency of many phytocompounds, from essential oils to more complex mixtures extracted from different plants [5,6]. Compared to synthetic pesticides, phytochemical biopesticides present lower toxicity, are less persistent, and biodegradable [1,7]. Phytochemical biopesticides, such as nicotine, rotenone, pyrethrins and sabadilla alkaloids, are some examples of the first series of botanical pesticides used. However, due to either their stability or toxicity and adverse effects on human health, their use has been restricted, and despite an increasing application, these remain less than 5% of the total pesticide use [8].

Phytochemical biopesticides comprise a series of secondary metabolites that act in a synergistic way, which strongly reduces the chances for insect pests to develop resistance. Literature has covered in detail many plant species and their use as biopesticides.

*Punica granatum* L. 1753, *Phytolacca americana* L. 1753, *Glandora prostrata* Loisel, 1844, *Ulex europaeus* L. 1753, *Tagetes patula* L. 1753, *Camellia japonica L.* 1753 and *Ruta graveolens* L. 1753 displayed a huge range of biological activities [3,9,10,11,12,13,14,15,16,17,18,19], including antibacterial (e.g., *P. granatum*) [9], antiviral (e.g., *P. granatum*, *C. japonica*) [10,11], antifungal (e.g., *P. granatum*, *T. patula*, *C. japonica*) [12], nematicide (*T. patula*) [13], herbicide (*U. europaeus*) and insecticide (e.g., *T. patula* and *R. graveolens*) [14,15,16,17,18,19].

The chemical composition and bioactivity of *R. graveolens* leaves were studied and tested against the maize weevil. The insecticidal and repellent properties of *R. graveolens* were analyzed and evaluated in contact and vapor forms, with a total of 45 compounds detected by chromatographic techniques, most of which were 2-ketones. The research carried out to date supports the evidence that *R. graveolens* leaves contain bioactive secondary plant metabolites, which justify its use against *S*. *zeamais* in storage pest management programs [20]. Lee et al. isolated essential oils from the flowers and leaves of *R. graveolens*, which were evaluated using fumigant and contact toxicity bioassays against store food pests, supporting the eco-potential of using *R. graveolens* essential oil and its major constituents in the management of stored pests [21,22].

Despite the reported activity against pests, several plant compounds exhibited some limitations in their application, due to their easy degradation and high volatility. Nanoencapsulation techniques arise as suitable strategies to allow for the preservation and controlled release of plant components [23,24]. Specifically, lipid-based nanosystems have been widely used as vehicles for bioactives in cosmetic and pharmaceutical industries [25,26], as well as for the encapsulation of plant extracts with insecticidal activity [27,28], being a promising route for a safe application of pesticides [29]. Plant extracts have also been encapsulated in cyclodextrins [24] and polymeric nanoparticles, the latter including alginate [30], gum arabic/maltodextrin [31], gelatin [32], polyvinylalcohol (PVA) [33], poly(dl-lactide-*co*-glycolide) (PLGA) [34] and chitosan nanostructures [35,36].

In this work, extracts from seven plant species, *P. granatum* (pomegranate), *P. americana* (American pokeweed), *G. prostrata* (shrubby gromwell), *U. europaeus* (gorce), *T. patula* (French marigold), *C. japonica* (camellia) and *R. graveolens* (rue or herb-of-grace), were isolated using water/ethanol and dichloromethane as solvents. The obtained extracts were evaluated as potential insecticides, through the assessment of their biological activity against *Sf9* insect cell lines, compared with a commercial synthetic pesticide. Considering the insecticidal activity exhibited by the *R. graveolens* dichloromethane extract, encapsulation assays in chitosan nanoemulsions and lipid nanosystems were carried out, for the future development of green insecticide nanoformulations.

## 2. Results and Discussion

### 2.1. Plant Extracts

A series of seven plant species were selected, taking into account their production in the country, endemic characteristics, the information of literature, as well as their unexploited potential in the field of bioinsecticides. After the harvest, the parts of plants/fruits that were intended to be studied were separated, namely fruit peel in the case of *P. granatum* (pomegranate); berries in the case of *P. americana* (American pokeweed); flowers and leaves in the case of *G. prostrata* (shrubby gromwell), *U. europaeus* (gorce), *R. graveolens* (rue or herb-of-grace), and *T. patula* (French marigold)—in the former, flowers (separated from the receptacle) and leaves were extracted separately—and leaves in the case of *C. japonica*. 

The preparation of the plant material included drying under sunlight (20–25 °C, 45 days) (peel of *P. Granatum*), the lyophilization (berries of *P. americana*, leaves and flowers of *T. patula*), or drying in an oven (40–45 °C, 3 days), followed in both cases by grounding with a shredder to obtain the corresponding powder, which was passed through a sieve until a particle size ≤ 900 μm was obtained. Furthermore, these materials were subjected to Soxhlet extraction with solvent systems of different polarities, namely in a polar system, using a water/ethanol solution (1:1), and in a less polar one, using dichloromethane, for 9 or 4 h, respectively. The solvents were removed by evaporation under reduced pressure (dichloromethane extracts) followed by lyophilization (aqueous ethanolic extracts). In order to remove the chlorophyll content, dichloromethane extracts were passed through a Chromabond C18 column, using methanol as eluent, followed by solvent evaporation. The 22 extracts thus obtained (11 in water/ethanol and 11 in dichloromethane, Table 1) were submitted to assays of cell viability using the insect cell line *Spodoptera frugiperda* (*Sf9*), to assess their capacity as potential bioinsecticides.

### 2.2. Evaluation of the Activity Against Sf9 Cells

**Figure 1:**
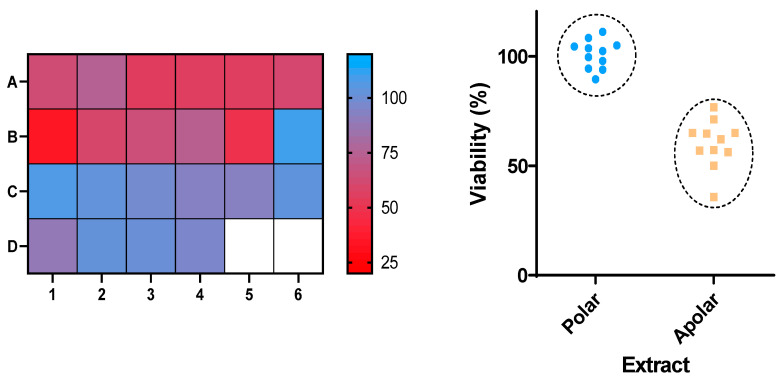
(**Left**): Results of the viability (%) of insect cells after treatment with the indicated samples. (**Right**): Comparison of the viability of cells treated with non-polar (**A1**–**B5**) vs. polar (**B6**–**C4**) extracts.

Given the high number of samples under study (22), we were interested in knowing which samples were the most toxic towards the *Sf9* cell line. To this end, all extracts were evaluated at the same concentration (100 µg/mL) for their impact in the viability of these cells after 24 h of incubation. For a clearer understanding, a heatmap of all results can be found in Figure 1 (left). Considering the results, there was a clear cluster of a group of samples that were active (red) and those that were mostly inactive (blue). The most active samples were, in fact, those arising from the use of non-polar solvents, as it can be seen in clearer detail in Figure 1 (right), when plotting the results from viability assays against the polarity of the extracts. This result can be explained by the fact that the use of nonpolar solvents allows the extraction of classes of natural products that are usually not extractable with highly polar solvents, such as water. As so, while polar solvents will extract molecules such as sugars, polyphenols and their glycosylated derivatives, the use of non-polar solvents widens the chemical diversity of the target molecules to include classes such as alkaloids, terpenes, coumarins, among others.

**Figure 2:**
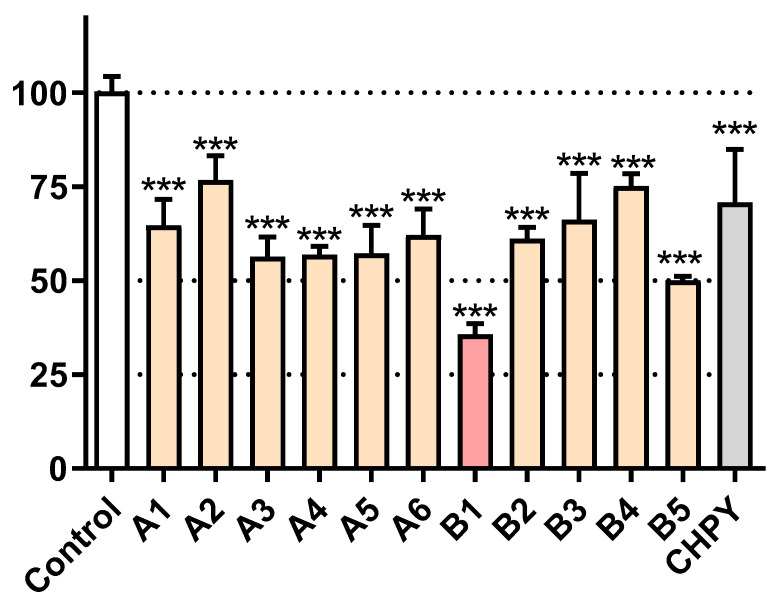
Viability of *Sf9* cells after exposure to the indicated samples at 100 µg/mL, for 24 h. CHPY = chlorpyrifos. *** *p* < 0.001. Identity of samples (**A1**–**A6** and **B1**–**B5**) as in Table 1.

Having reduced the number of samples under study, we were interested in knowing which species were triggering higher levels of toxicity. As seen in Figure 2, most samples reduced cell viability to the 50–75% range; however, the *R. graveolens* dichloromethane extract (**B1**, Table 1) was able to reduce the viability below 50%. For benchmarking purposes, we additionally tested the viability of *Sf9* cells after exposure to the commercial synthetic insecticide chlorpyrifos. As shown in Figure 2, this molecule elicited ca. 50% of viability loss, thus being less potent than **B1**. For this reason, the latter was selected for subsequent studies.

The UHPLC ESI-Q-TOF-MME analysis of *R. graveolens* extract revealed the presence of ten compounds, some of which show a molecular mass compatible with quinolin, benzopyran, and acridone derivatives (Table 2). A chromatogram of the extract is shown in the Supporting Information Figure S1.

### 2.3. Morphological Assessment

**Figure 3:**
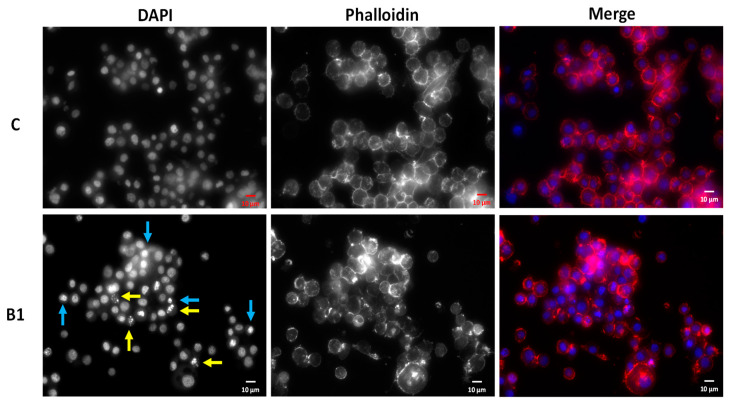
*Sf9* cells exposed to **B1** (100 µg/mL) for 24 h (S Plan Fluor ELWD 40 × DIC N1 objective). Cell morphology was evaluated using 4′,6-diamidino-2-phenylindole (DAPI, chromatin) and phalloidin (actin). Yellow arrow: chromatin fragmentation; Blue arrow: chromatin condensation. (**C**)—control. Color blind-friendly image in Supporting Information Figure S2.

In light of the pronounced impact of **B1** in *Sf9* cell viability, we were interested in understanding the underlying effect of the extract. To this end, the control and **B1**-treated cells have been imaged in order to assess putative differences in overall cell morphology and chromatin. As shown in Figure 3, **B1** elicits a significant degree of chromatin condensation (blue arrows) and even fragmentation (yellow arrows), although overall cell integrity is maintained. This result suggests that **B1** may be eliciting the loss of cell viability by triggering an organized process of cell death, in which morphological traits are compatible to those described here.

### 2.4. Nanoencapsulation Studies

The size and size distribution of the chosen nanoencapsulation systems was determined by dynamic light scattering (DLS), Table 3. The liposomes prepared by the ethanolic injection method have a smaller size than the ones prepared by thin film hydration but exhibit a slightly higher polydispersity. The chitosan nanostructures (prepared by ionic gelation) are the encapsulation systems with the lowest hydrodynamic diameter. The sizes were maintained constant for two weeks, with no significant change in polydispersity, proving the stability of the prepared nanosystems.

Liposomes have been described as ideal bioactive delivery systems, due to their biocompatibility and their ability to carry both hydrophilic (in the aqueous lumen) and hydrophobic (in the lipid bilayer) payloads [40]. The results in Table 3 are in accordance with the ones reported in the literature for lecithin-based liposomal systems prepared by ethanolic injection [41,42] and thin film hydration [43,44], the latter being slightly larger. Chitosan nanostructures have also been described as advantageous for the encapsulation of pesticides and natural extracts [45,46,47,48]. The hydrodynamic diameter and polydispersity obtained for chitosan nanocapsules (Table 3) are also in accordance with the reported results for a similar preparation method [45].

The encapsulation efficiencies of the extract with the best insecticidal activity **B1** in soybean liposomes and chitosan nanoemulsions are presented in Table 4.

The encapsulation efficiencies were generally high (above 70%), especially in liposomes prepared by the ethanolic injection method and in chitosan nanostructures.

**Figure 4:**
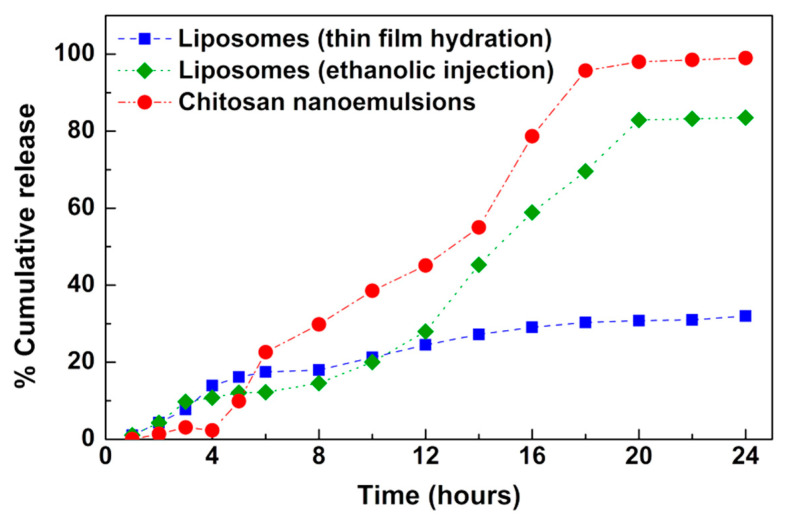
Release profiles of sample **B1** from the liposomes and chitosan nanostructures.

The release of the encapsulated extract was followed for 24 h at room temperature (25 °C) towards phosphate buffer, pH = 7.3 (Figure 4). The results evidence that chitosan nanostructures are suitable for a faster and complete release, while liposomes allow a delayed release. The difference in the release profiles obtained from liposomes prepared by the two techniques (thin film hydration and ethanolic injection) may be related with the hydrophobic character of this extract, as ethanolic injection is more advantageous for this type of bioactives [41].

The experimental release profiles were analyzed by a modified Korsmeyer–Peppas model [49] and the results are summarized in Table 5 (the fittings are shown in Supporting Information Figure S3).

Considering that the nanoencapsulation systems are spherical, the *n* value in the range 0.43 < *n* < 0.85 suggests that the release profiles from liposomes prepared by thin film hydration can be mainly ascribed to diffusion and swelling, i.e., both contributions have comparable rates [49]. The samples marked with * in Table 5 were subjected to a different analysis, for which the release is initially assumed to be associated with the super case-ii transport mechanism, where erosion, relaxation and diffusion are responsible for extract release. Despite the reasonable fitting of the model to the experimental data obtained in liposomes prepared by injection and chitosan nanostructures (as implied by the coefficients of determination), it does not properly explain the data from a qualitative point of view. Therefore, the release profiles were also fitted to the Gompertz empirical function (Table 6), defined as

$$X_{t}=X_{max}e^{-ae^{b\log_{10}t}}$$

where $X_{t}$ and $X_{max}$ are the dissolved fraction percentages at time *t* and its maximum, a is a shape parameter and b is the dissolution rate per unit of time [50]. The fittings to this model are also generally good (see Supporting Information Figure S4). Although the model does not provide mechanistic information, the larger *b* values of the samples in chitosan indicate faster release kinetics, while liposomes obtained by hydration seem to be more effective in ensuring a slower release.

The results obtained herein indicate that lipid-based and chitosan nanoencapsulation systems allow high encapsulation efficiencies of the extracts with insecticidal activity and provide different release rates. This represents a proof-of-concept to future developments of bioinsecticide nanoformulations using the most active extracts.

## 3. Experimental Section

### 3.1. Chemicals and Reagents

Ethanol, methanol, dichloromethane, isopropanol, DMSO, acetic acid and tetrahydrofuran were purchased from Merck KGaA (Darmstadt, Germany). Chromabond C18 column was purchased from Sigma-Aldrich (St. Louis, MO, USA). Trypan blue and 3-(4,5-dimethylthiazolyl-2)-2,5-diphenyltetrazolium bromide (MTT) were obtained from Sigma-Aldrich (St. Louis, MO, USA). Dulbecco’s modified eagle medium (DMEM), Hank’s balanced salt solution (HBSS), fetal bovine serum (FBS), penicillin–streptomycin solution (penicillin 5000 units/mL and streptomycin 5000 μg/mL) and 0.25% trypsin-EDTA were obtained from GIBCO, Invitrogen™ (Grand Island, NY, USA).

### 3.2. Plant Material

The seven species of plants were harvested in the North of Portugal, namely in Melgaço (42.1144° N, 8.2580° W) (*P. granatum*, *P. americana*, *G. prostrata*, *U. europaeus*, *T. patula*, *C. japonica* red) and Guimarães (41.4425° N, 8.2918° W) (*R. graveolens*), at the flowering stage in the case of *G. prostrata*, *U. europaeus* and *T. patula* in March 2019 (*G. prostrata*, *U. europaeus*) or October 2019 for the remaining plants. The plant material included fruit peel (*P. granatum*), berries (*P. americana*), flowers and leaves (*G. prostrata*, *U. europaeus*, and *T. patula*), as well as only leaves (*C. japonica* and *R. graveolens*). In the case of *T. patula,* the leaves and yellow, orange and red flowers (separated from the receptacle) were used in separate experiments. At least three specimens of each species were collected.

The pomegranate peel (*P. granatum*) was dried under sunlight at 20–25 °C during 45 days. The berries of *P. americana*, as well as the leaves and flowers of *T. patula*, were frozen at −20 °C until lyophilization in an Alpha 1-4 ld Plus–Christ freeze dryer (Martin Christ Gefriertrocknungsanlagen GmbH, Osterode am Harz, Germany). The leaves and flowers of the remaining species were dried in an oven at 40–45 °C during 3 days.

After drying or lyophilization, the vegetable matter was ground with a shredder, and the resulting powder passed through a sieve until a particle size ≤ 900 μm was obtained. The material was stored under vacuum for further use.

### 3.3. Plant Materials Extraction

The dried or lyophilized plant materials of *P. granatum*, *P. americana*, *G. prostrata*, *U. europaeus*, *T. patula*, *C*. *japonica*, *R. graveolens* (8 g) were subjected to Soxhlet extraction with water/ethanol (1:1) (50 mL) or dichloromethane (50 mL) for 9 or 4 h, respectively.

The organic solvents were removed by evaporation under reduced pressure at 40 °C (Rotavapor^®^ R-210, BÜCHI Labortechnik AG, Flawil, Switzerland), followed by lyophilization (Alpha 1-4 ld Plus–Christ freeze dryer, Martin Christ Gefriertrocknungsanlagen GmbH, Osterode am Harz, Germany) in the case of extractions with water/ethanol (1:1). The resulting lyophilized extracts were stored under vacuum for further use. The evaporated extracts of dichloromethane were passed through a Chromabond C18 column, using methanol as eluent, for the removal of chlorophylls. After the solvent evaporation under vacuum at 35 °C, the resulting extracts were stored at −20 °C for further use.

### 3.4. Cell Culture

*Sf9* (*Spodoptera frugiperda*) cells were from ATCC/LGC Standards (Spain) maintained as a suspension culture and cultivated in Grace’s medium with 10% FBS and antibiotics at 28 °C. Cells were used in the experiments while in the exponential phase of growth. HaCaT (human keratinocytes) cells were cultured in DMEM supplemented with 10% FBS and 1% penicillin/streptomycin at 37 °C, in a humidified atmosphere of 5% CO_2_.

### 3.5. Assessment of Viability

For the assessment of viability, a resazurin-based method was used, as previously described [51]. *Sf9* cells were plated at a density of 3 × 10^4^ cells/well, incubated for 24 h and then exposed to the molecules under study for 24 h. After this period, a commercial solution of resazurin was added (1:10) and the kinetic reaction of fluorescence increase was monitored. An incubation time of 30 min was used.

### 3.6. Morphological Assessment

For morphological studies, the cells were cultured in 96-well plates, as described above for viability experiments, in the presence of the samples under study. After incubation, the cells were washed with HBSS and fixed in 10% formalin solution for 30 min, at room temperature. DAPI (0.25 µg/mL) and CF543 (5 U/mL) were added and the cells were stained for 25 min at room temperature and washed with HBSS, as described before [52,53].

Images were acquired in an inverted Eclipse Ts2R-FL (Nikon, Amsterdam) microscope equipped with a Retiga R1 camera and a S Plan Fluor ELWD 20× DIC N1 objective. Images were analyzed with Fiji [54]. For quantitative parameters, the Fiji’s Cell Counter plugin was used.

### 3.7. Nanoencapsulation Studies

#### 3.7.1. Liposomes Preparation

Liposomes were prepared by both the ethanolic injection [41] and thin film hydration [43] methods, using a commercial lipid mixture for food industry, soy lecithin (Sternchemie), containing (% mol/mol) 22% phosphatidylcholine, 20% phosphatidylethanolamine, 20% phosphatidylinositol, and 10% phosphatidic acid as main components, with a concentration of 1 × 10^−3^ M. In the ethanolic injection method [41], 2 × 10^−3^ g/mL extract was added to the lipid mixture in ethanol and under vortexing, the mixture was slowly added to 3 mL of ultrapure water.

For thin film hydration method [43], a lipidic film was obtained from the evaporation of ethanolic lipid solution and the addition of the extract, followed by hydration, bath sonication, and extrusion (in a Lipex™ Extruder, Northern Lipids, Burnaby, BC, Canada) through polycarbonate membranes (200 nm pore size).

#### 3.7.2. Chitosan Nanocapsules Preparation

The preparation of chitosan nanocapsules was performed by ionic gelation, as described by Chauhan et al. [45]. Briefly, 2.8 mg of chitosan was dissolved in Tris-HCL buffer (pH = 7.3) and acetic acid (1% *v*/*v*) was added. The pH was raised until 4.6–4.8 by the addition of NaOH 10 M. Then, 14 μL of Tween^®^ 80 surfactant was added and the obtained dispersion was filtered. Then, the plant extract in ethanol was added (1% *v*/*w* relative to chitosan). Twenty milliliters of sodium tripolyphosphate (TPP) aqueous solution (0.25% *w*/*v*) was slowly added to 60 mL of chitosan solution under continuous magnetic stirring. Nanocapsules were then centrifuged at 20,000 g for 30 min and the supernatant was discarded.

#### 3.7.3. Size Measurements

The mean hydrodynamic diameter and size distribution (polydispersity index) of the prepared nanoencapsulation systems were measured using dynamic light scattering (DLS) equipment NANO ZS Malvern Zetasizer (Malvern Panalytical Ltd., Malvern, UK) at 25 °C, using a He–Ne laser of λ = 632.8 nm and a detector angle of 173°. Five independent measurements were performed for each sample.

#### 3.7.4. Encapsulation Efficiencies and Release Assays

Dilutions of the selected extracts, in the range of concentrations 5 × 10^−3^–1 × 10^−1^ mg /mL were carried out to determine the calibration curve (absorbance vs. concentration) and calculate the encapsulation efficiency. Loaded nanosystems were subjected to centrifugation at 11,000 rpm for 60 min using Amicon^®^ Ultra centrifugal filter units 100 kDa. Then, the filtrate (containing the non-encapsulated compound) was pipetted out and its absorbance was measured, allowing the determination of compound concentration using a calibration curve previously obtained in the same solvent (Figure S5 in Supporting Information). Absorption spectra were performed in a Shimadzu UV-3600 Plus UV-Vis-NIR spectrophotometer (Shimadzu Corporation, Kyoto, Japan).

Three independent measurements were performed for each system. The encapsulation efficiency, *EE*(%), was obtained through Equation (1):

*EE(%) = (Total amount − Amount of non-encapsulated extract)/(Total amount) × 100* (1)

Release assays to phosphate buffer (pH = 7.3) were performed during 24 h, using dialysis membranes and were done in triplicate. The systems were maintained under magnetic stirring at 25 ± 0.5 °C, and the solutions were kept covered to prevent evaporation during the assay. The obtained experimental release profiles were analyzed by a modified Korsmeyer–Peppas model [49], which accounts for the latency time:

(2) $$\frac{M_{\left(t-l\right)}}{M_{\infty }}=K_{s}{\left(t-l\right)}^{n}$$

where *M* is the quantity of the released agent, *K*_s_ is the rate constant, *t* is the time, l is the latency time and *n* is the transport exponent. When *n* < 0.45, the release mechanism is diffusion-controlled (Fickian release), 0.45 < *n* < 0.89 indicates a combination of diffusion and erosion drug release (non-Fickian release), 0.89 < *n* < 1 indicates a relaxation-controlled release, and if *n* > 1, the release is controlled by swelling and material relaxation [49].

## 4. Conclusions

In this work, a bioactive extract towards insect cells was identified from a pool of over twenty extracts from seven different plant species. The non-polar extract from *R. graveolens* was able to reduce cell viability over 50% at the concentration of 100 µg/mL, while eliciting morphological changes typically found in processes of regulated cell death. Importantly, the effect of this extract exceeded that of the commercial insecticide chlorpyrifos at the same concentration. LC–MS analysis allowed the identification of the major phytochemicals of the extract, which may be responsible for this insecticide effect either alone or in association.

The nanoencapsulation systems tested allowed the release of the biologically active extract with different release rates. Overall, the results of this work are promising for future developments of biopesticide formulations.

## Figures and Tables

**Table 1:** Extracts of various species obtained using two different solvent systems and studied against *Sf9*.

Entry	Specie	Part of the Plant	Solvent	Code
1	*Phytolacca americana*	Leaves	DCM	A1
2	*Phytolacca americana*	Berries	DCM	A2
3	*Phytolacca americana*	Berries	Water/ EtOH (1:1)	D2
4	*Phytolacca americana*	Leaves	Water/ EtOH (1:1)	D3
5	*Tagetes patula* (red flowers)	Leaves	DCM (F.1-3)	A3
6	*Tagetes patula* (yellow flowers)	Leaves	DCM (F.1-4)	A4
7	*Tagetes patula* (orange flowers)	Leaves	DCM (F.1-3)	A5
8	*Tagetes patula* (red flowers)	Flowers	DCM	A6
9	*Tagetes patula* (red flowers)	Leaves	Water/ EtOH (1:1)	B6
10	*Tagetes patula*	Flowers (red)	Water/ EtOH (1:1)	C1
11	*Tagetes patula* (yellow flowers)	Leaves	Water/ EtOH (1:1)	C2
12	*Tagetes patula* (orange flowers)	Leaves	Water/ EtOH (1:1)	C3
13	*Ruta graveolens*	Leaves	DCM	B1
14	*Ruta graveolens*	Leaves	Water/ EtOH (1:1)	D4
15	*Ulex europaeus*	Leaves and flowers	DCM	B2
16	*Ulex europaeus*	Leaves and flowers	Water/ EtOH (1:1)	C5
17	*Glandora prostrata*	Leaves and flowers	DCM	B3
18	*Glandora prostrata*	Leaves and flowers	Water/ EtOH (1:1)	D1
19	*Punica granatum*	Fruit peel	DCM	B4
20	*Punica granatum*	Fruit peel	Water/ EtOH (1:1)	C4
21	*Camellia japonica*	Leaves	DCM	B5
22	*Camellia japonica*	Leaves	Water/ EtOH (1:1)	C6

**Table 2:** Mass spectrometry data and putative identification for the *R. graveolens* extract. The peak mentioned is the chromatogram peak number shown in the Supporting Information Figure S2.

Peak	RT	M^+^	Compound	Reference
10	19.477	279	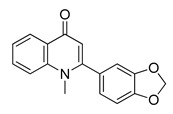 Graveoline	[37]
11	20.455	259	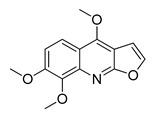 Skimmianin	[37]
11	20.455	261	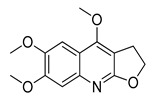 4,6,7-Trimethoxy-furo[2,3-*b*]quinoline	[38]
16	23.932	216	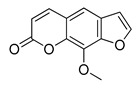 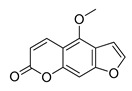 8-Methoxypsoralen 5-Methoxypsoralen	[39]
17	24.082	229	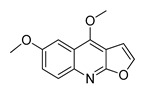 Pteleine	[37]
18	33.006	314	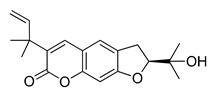 Chalepin	[37]
21	38.681	356	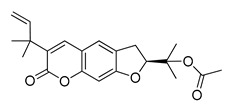 Rutamarin	[37]
20	37.305	271	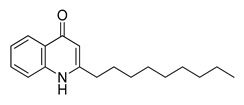 2-*N*-Nonyl-4-quinolone	[38]
21	38.681	357	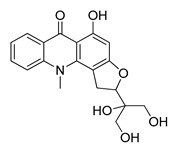 Gravacridonetriol	[37]

**Table 3:** Size (hydrodynamic diameter) and the polydispersity of soybean liposomes and chitosan nanostructures determined by dynamic light scattering (DLS) (SD: standard deviation from five independent measurements).

System	Size ± SD (nm)	PDI ± SD
Liposomes (ethanolic injection)	121 ± 28	0.256 ± 0.08
Liposomes (thin film hydration)	203 ± 17	0.185 ± 0.05
Chitosan nanostructures	118 ± 23	0.203 ± 0.02

**Table 4:** Encapsulation efficiencies (EE% ± SD) of the extract with the best insecticidal activity in liposomes and chitosan nanoemulsions (SD: standard deviation of three independent measurements).

System	Sample B1
Liposomes (ethanolic injection)	93.3 ± 6
Liposomes (thin film hydration)	73.0 ± 9
Chitosan nanoemulsions	94.0 ± 2

**Table 5:** Release parameters of the Korsmeyer–Peppas model fitted to the release profiles (*R* is the coefficient of determination).

Nanosystem	*K*s	*n*	*l*	$R^{2}$
Liposomes (thin film hydration)	0.07	0.50	0.9	0.98
Liposomes (ethanolic injection) Liposomes (ethanolic injection) *	0.01	1.39	0	0.98
	0.0045	1.74	0	0.99
Chitosan nanostructures Chitosan nanostructures *	0.04	1.07	2.13	0.98
	0.0314	1.21	2.11	0.99

**Table 6:** Release parameters obtained from the fitting of the Gompertz model to the release profiles.

Nanosystem	$X_{max}\;$	*a*	*B*	$R^{2}\;$
Liposomes (thin film hydration)	49.38	3.61	1.55	0.99
Liposomes (ethanolic injection)	100	680.48	5.99	0.99
Chitosan nanostructures	100	163.23	5.34	0.97

## Supplementary Materials

The following are available online, Figure S1: HPLC–DAD chromatogram of *Ruta graveolens* extract (mobile phase: A: 0.1% FA in H_2_O; B: 0.1% FA in ACN; injection: 20 µL; oven 30 °C; DAD: 410, 300 and 250 nm; column: ZORBAX ECLIPSE XDB-C18, 4.6 × 150 mm, 5 micron). Figure S2: *Sf9* cells exposed to **B1** (100 µg/mL) for 24 h (S Plan Fluor ELWD 40× DIC N1 objective). Cell morphology was evaluated using DAPI (chromatin) and phalloidin (actin). Yellow arrow: chromatin fragmentation; blue arrow: chromatin condensation. **C**: control. Figure S3: Fittings to the Korsmeyer–Pepper model. Lipo-thf: liposomes (thin film hydration); Lipo-inj: liposomes (ethanolic injection); Cht: chitosan nanostructures. Figure S4: fittings to Gompertz model. Lipo-thf: liposomes (thin film hydration); Lipo-inj: liposomes (ethanolic injection); Cht: chitosan nanostructures. Figure S5: **A.** UV–Visible absorption spectrum of the extract of *Ruta graveolens* L. **B.** Calibration curve (absorbance vs. concentration) for the determination of encapsulation efficiencies.

## References

[1] Seiber J.N., Coats J., Duke S.O., Gross A.D. (2014). Biopesticides: State of the art and future opportunities. J. Agric. Food Chem..

[2] Rob M.M., Hossen K., Iwasaki A., Suenaga K., Kato-Nogushi H. (2020). Phytotoxic activity and identification of phytotoxic substances from *Schumanniathus dichotomus*. Plants.

[3] Pavela R., Maggi F., Iannareli R., Benelli G. (2019). Plant extracts for developing mosquito larvicides: From laboratory to the field, with insights on the modes of action. Acta Trop..

[4] Yang G.-Z., Zhang J., Peng J.-W., Zhang Z.-J., Zhao W.-B., Wang R.-X., Ma K.-Y., Li J.-C., Liu Y.-Q., Zhao Z.-M. (2020). Discovery of luotonin A analogues as potent fungicides and insecticides: Design, synthesis and biological evaluation inspired by natural alkaloid. Eur. J. Med. Chem..

[5] Ebadollahi A., Ziaee M., Palla F. (2020). Essential oils extracted from different species of the *Lamiaceae* plant family as prospective bioagents against several detrimental pests. Molecules.

[6] Magierowicz K., Górska-Dabrik E., Golan K. (2020). Effects of plant extracts and essential oils on the behavior of *Acrobasis advenella* (Zinck.) caterpillars and females. J. Plant Dis. Prot..

[7] Walia S., Saha S., Tripathi V., Sharma K.K. (2017). Phytochemical biopesticides: Some recent developments. Phytochem. Rev..

[8] Sparks T.C., Lorsbach B.A., Gross A.D., Ozoe Y., Coats J.R. (2017). Agrochemical discovery—Building the next generation of insect control agents. Advances in Agrochemicals: Ion Channels and G Protein-Coupled Receptors (GPCRs) as Targets for Pest Control.

[9] Singh R.P., Chidambara Murthy K.N., Jayaprakasha G.K. (2002). Studies on the antioxidant activity of pomegranate (Punica granatum) peel and seed extracts using in vitro models. J. Agric. Food Chem..

[10] Singh B., Singh J.P., Kaur A., Singh N. (2018). Phenolic compounds as beneficial phytochemicals in pomegranate (*Punica granatum* L.) peel: A review. Food Chem..

[11] Yoon I.S., Park D.H., Kim J.E., Yoo J.C., Bae M.S., Oh D.S., Cho S.S. (2017). Identification of the biologically active constituents of *Camellia japonica* leaf and anti-hyperuricemic effect in vitro and in vivo. Int. J. Mol. Med..

[12] Navabi S.M., Ebrahimzadeh M.A., Navabi S.F., Bahramian F. (2009). In vitro antioxidant activity of *Phytolacca americana* berries. Pharmacologyonline.

[13] Ismail T., Sestili P., Akhtar S. (2012). Pomegranate peel and fruit extracts: A review of potential anti-inflammatory and anti-infective effects. J. Etnopharmacol..

[14] Ismail T., Sestili P., Akhtar S. Pomegranate Peel and Fruit Extracts: A Review of Potential Plants for A Future. *Tagetes Patula* French Marigold, Dwarf French Marigold PFAF Plant Database. https://pfaf.org/user/Plant.aspx?LatinName=Tagetes+patula.

[15] Pardo-Muras M., Puig C.G., Lopez-Nogueira A., Cavaleiro C., Pedrol N. (2018). On the bioherbicide potential of *Ulex europaeus* and *Cytisus scoparius*: Profiles of volatile organic compounds and their phytotoxic effects. PLoS ONE.

[16] Fabrick J.A., Yool A.J., Spurgeon D.W. (2020). Insecticidal activity of marigold *Tagetes patula* plants and foliar extracts against the hemipteran pests, *Lygus hesperus* and *Bemisia tabaci*. PLoS ONE.

[17] Chaaban S.B., Hamdi S.H., Mahjoubi K., Jemâa J.M.B. (2019). Composition and insecticidal activity of essential oil from *Ruta graveolens*, *Mentha pulegium* and O*cimum basilicum* against *Ectomyelois ceratoniae* Zeller and *Ephestia kuehniella* Zeller (Lepidoptera: Pyralidae). J. Plant Dis. Prot..

[18] Semerdjieva I.B., Burducea M., Astatkie T., Zheljazkov V.D., Dincheva I. (2019). Essential oil composition of *Ruta graveolens* L. fruits and *Hyssopus officinalis Subsp. aristatus* (Godr.) Nyman biomass as a function of hydrodistillation time. Molecules.

[19] Plants for a Future. *Ruta graveolens Rue*, Common rue, Herb of Grace, Garden Rue PFAF Plant Database. https://pfaf.org/user/Plant.aspx?LatinName=Ruta+graveolens.

[20] Perera A., Karunaratne M., Chinthaka S. (2017). Biological activity and secondary metabolite profile of *Ruta graveolens* leaves against maize weevil infestations. J. Entomol. Zool. Stud..

[21] Jeon J.-H., Lee S.-G., Lee H.-S. (2015). Isolation of insecticidal constituent from *Ruta graveolens* and structure-activity relationship studies against stored-food pests (Coleoptera). J. Food Prot..

[22] Perera A.G.W.U., Karunaratne M.M.S.C., Chinthaka S.D.M. (2019). Qualitative determination, quantitative evaluation and comparative insecticidal potential of *Ruta Graveolens* essential oil and its major constituents in the management of two stored pests Sitophilus Zeamais (*Coleoptera: Curculionidae*) and Corcya Cephalonica (Lepidoptera: Pyralidae). Sustain. Dev. Res..

[23] El Asbahani A., Miladi K., Badri W., Sala M., Addi E.H.A., Casabianca H., El Mousadik A., Hartmann D., Jilale A., Renaud F.N. (2015). Essential oils: From extraction to encapsulation. Int. J. Pharm..

[24] Pinho E., Grootveld M., Soares G., Henriques M. (2014). Cyclodextrins as encapsulation agents for plant bioactive compounds. Carbohydr. Polym..

[25] Bulbake U., Doppalapudi S., Kommineni N., Khan W. (2017). Liposomal formulations in clinical use: An updated review. Pharmaceutics.

[26] Shi F., Zhao J.-H., Liu Y., Wang Z., Zhang Y.-T., Feng N.-P. (2012). Preparation and characterization of solid lipid nanoparticles loaded with frankincense and myrrh oil. Int. J. Nanomed..

[27] Varona S., Marti’n A., Cocero M.J. (2011). Liposomal incorporation of lavandin essential oil by a thin-film hydration method and by particles from gas-saturated solutions. Ind. Eng. Chem. Res..

[28] Liolios C.C., Gortzi O., Lalas S., Tsaknis J., Chinou I. (2009). Liposomal incorporation of carvacrol and thymol isolated from the essential oil of *Origanum dictamnus* L. and in vitro antimicrobial activity. Food Chem..

[29] Nuruzzaman M., Rahman M.M., Liu Y., Naidu R. (2016). Nanoencapsulation, nano-guard for pesticides: A new window for safe application. J. Agric. Food Chem..

[30] Hosseini S.M., Hosseini H., Mohammadifar M.A., Mortazavian A.M., Mohammadi A., Khosravi-Darani K., Shojaee-Aliabadi S., Dehghan S., Khaksar R. (2003). Incorporation of essential oil in alginate microparticles by multiple emulsion/ionic gelation process. Int. J. Biol. Macromol..

[31] López A., Castro S., Andina M.J., Ures X., Munguía B., Llabot J.M., Elder H., Dellacassa E., Palma S., Domínguez L. (2014). Insecticidal activity of microencapsulated *Schinus molle* essential oil. Ind. Crops. Prod..

[32] Sutaphanit P., Chitprasert P. (2014). Optimisation of microencapsulation of holy basil essential oil in gelatin by response surface methodology. Food Chem..

[33] Leimann F.V., Gonçalves O.H., Machado R.A.F., Bolzan A. (2009). Antimicrobial activity of microencapsulated lemongrass essential oil and the effect of experimental parameters on microcapsules size and morphology. Mater. Sci. Eng. C.

[34] Iannitelli A., Grande R., Di Stefano A., Di Giulio M., Sozio P., Bessa L.J., Laserra S., Paolini C., Protasi F., Cellini L. (2011). Potential antibacterial activity of carvacrol-loaded poly(DL-lactide-co-glycolide) (PLGA) nanoparticles against microbial biofilm. Int. J. Mol. Sci..

[35] Hosseini S.F., Zandi M., Rezaei M., Farahmandghavi F. (2013). Two-step method for encapsulation of oregano essential oil in chitosan nanoparticles: Preparation, characterization and in vitro release study. Carbohydr. Polym..

[36] Jamil B., Abbasi R., Abbasi S., Imran M., Khan S., Ihsan A., Javed S., Bokhari H., Imran M. (2016). Encapsulation of cardamom essential oil in chitosan nano-composites: In-vitro efficacy on antibiotic-resistant bacterial pathogens and cytotoxicity studies. Front. Microbiol..

[37] Ulubelen A., Ozturk M. (2006). Alkaloids and coumariuns from *Ruta* Species. Nat. Prod. Commun..

[38] Oliva A., Meepagala K.M., Wedge D.E., Harries D., Hale A.L., Aliotta G., Duke S.O. (2003). Natural fungicides from *Ruta graveolens* L. leaves, including a new quinolone alkaloid. J. Agric. Food Chem..

[39] Hale A.L., Meepagala K.M., Oliva A., Aliotta G., Duke S.O. (2004). Phytotoxins from the leaves of Ruta graveolens. J. Agric. Food Chem..

[40] Malam Y., Loizidou M., Seifalian A.M. (2009). Liposomes and nanoparticles: Nanosized vehicles for drug delivery in cancer. Trends Pharmacol. Sci..

[41] Jaafar-Maalej C., Diab R., Andrieu V., Elaissari A., Fessi H. (2010). Ethanol injection method for hydrophilic and lipophilic drug-loaded liposome preparation. J. Liposome Res..

[42] Pereira D.S.M., Cardoso B.D., Rodrigues A.R.O., Amorim C.O., Amaral V.S., Almeida B.G., Queiroz M.J.R.P., Martinho O., Baltazar F., Calhelha R.C. (2019). Magnetoliposomes containing calcium ferrite nanoparticles for applications in breast cancer therapy. Pharmaceutics.

[43] Zhang H. (2017). Thin-film hydration followed by extrusion method for liposome preparation. Methods Mol. Biol..

[44] Pradhan P., Giri J., Banerjee R., Bellare J., Bahadur D. (2007). Preparation and characterization of manganese ferrite-based magnetic liposomes for hyperthermia treatment of cancer. J. Magn. Magn. Mater..

[45] Chauhan N., Dilbaghi N., Gopal M., Kumar R., Kim K.H., Kumar S. (2017). Development of chitosan nanocapsules for the controlled release of hexaconazole. Int. J. Biol. Macromol..

[46] Esquerdo V.M., Dotto G.L., Pinto L.A.A. (2015). Preparation of nanoemulsions containing unsaturated fatty acid concentrate-chitosan capsules. J. Coll. Interface Sci..

[47] Mo F., Lin B., Lai F., Xu C., Zou H. (2016). A green modified microsphere of chitosan encapsulating dimethyl fumarate and cross-linked by vanillin and its application for litchi preservation. Ind. Eng. Chem. Res..

[48] Xiao Z., Xu Z., Zhu G. (2017). Production and characterization of nanocapsules encapsulated linalool by ionic gelation method using chitosan as wall material. Food Sci. Technol..

[49] Bruschi M. (2015). Strategies to Modify the Drug Release from Pharmaceutical Systems.

[50] Dash S., Murthy P.N., Nath L., Chowdhury P. (2010). Kinetic modelling on drug release from controlled drug delivery systems. Acta Pol. Pharm..

[51] Fernandes M.J.G., Castanheira E.M.S., Fortes A.G., Pereira R.B., Pereira D.M., Gonçalves M.S.T. (2020). New eugenol derivatives and their selective toxicity towards insect cells: Synthesis and biological assessment. Int. J. Mol. Sci..

[52] Ribeiro V., Andrade P.B., Valentão P., Pereira D.M. (2019). Benzoquinones from *Cyperus* spp. trigger IRE1α-independent and PERK-dependent ER stress in human stomach cancer cells and are novel proteasome inhibitors. Phytomedicine.

[53] Chouiter I., Boulebd H., Pereira D.M., Valentão P., Andrade P., Belfaitah A., Silva A. (2020). Chalcone-type compounds and 2-pyrazoline derivatives bearing an imidazole moiety: Synthesis and caspase-dependent anticancer activity. Future Med. Chem..

[54] Schindelin J., Arganda-Carreras I., Frise E., Kaynig V., Longair M., Pietzsch T., Preibisch S., Rueden C., Saalfeld S., Schmid B. (2012). Fiji: An open-source platform for biological-image analysis. Nat. Methods.

