# Supplementary material for: Cytotoxic Plant Extracts towards Insect Cells: Bioactivity and Nanoencapsulation Studies for Application as Biopesticides
Source: Molecules. 2020 Dec 11;25(24):5855. doi: 10.3390/molecules25245855 (PMC7764084; doi:10.3390/molecules25245855)
Supplement: Supplementary file 1 [file molecules-25-05855-s001.pdf]

Supplementary Material

Cytotoxic Plant Extracts towards Insect Cells: Bioactivity and Nanoencapsulation Studies for Application as Biopesticides

Ana I. F. Lopes[1,2], Mariana Monteiro[1,2], Ana R. L. Araújo[1], A. Rita O. Rodrigues [2], Elisabete M. S. Castanheira[2], David M. Pereira[3], Pedro Olim[3], A. Gil Fortes[1], M. Sameiro T. Gonçalves[1]*

1 Centre of Chemistry, Department of Chemistry, University of Minho, Campus of Gualtar, 4710-057 Braga, Portugal; pg35027@alunos.uminho.pt (A.I.F.L.); pg31434@alunos.uminho.pt (M.M.); rita_leite3@hotmail.com (A.R.L.A.); gilf@quimica.uminho.pt (A.G.F.)

2 Centre of Physics, Department of Physics, University of Minho, Campus of Gualtar, 4710-057 Braga, Portugal; ritarodrigues@fisica.uminho.pt (A.R.O.R.); ecoutinho@fisica.uminho.pt (E.M.S.C.)

3 REQUIMTE/LAQV, Laboratory of Pharmacognosy, Department of Chemistry, Faculty of Pharmacy, University of Porto, R. Jorge Viterbo Ferreira, 228, 4050-313 Porto, Portugal; dpereira@ff.up.pt (D.P.P.); up201706671@ff.up.pt (P.O.)

* Correspondence: msameiro@quimica.uminho.pt; Tel.: +351253604372

Sample Chromatograms

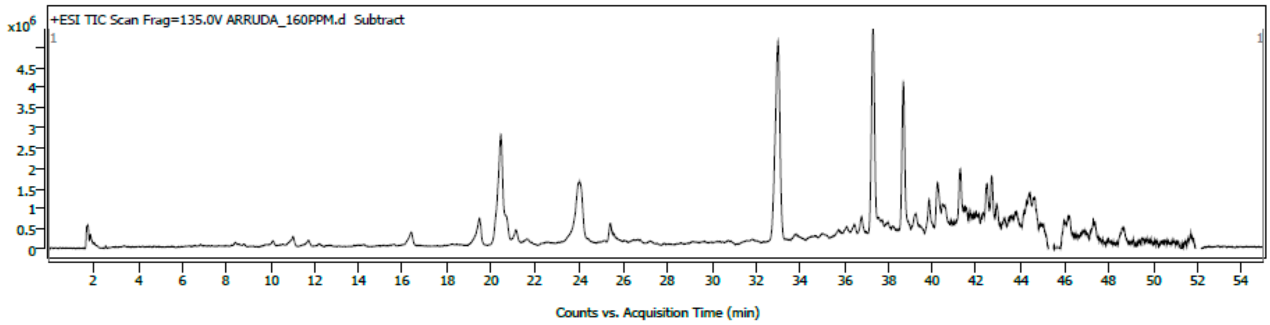

Chromatogram Peaks

Peak	Start	RT	End	Height	Area	Area %
1	1.630	1.683	1.731	305043	1108248	2.33
2	1.731	1.741	1.824	106389	280341	0.59
3	8.337	8.413	8.491	43496	201635	0.42
4	9.962	10.120	10.208	21909	151154	0.32
5	10.709	11.040	11.156	89258	949674	2.00
6	11.511	11.711	11.808	19552	160523	0.34
7	14.122	14.249	14.341	31712	201443	0.42
8	16.076	16.384	16.548	113671	1340392	2.82
9	18.101	18.237	18.329	18640	143316	0.30
10	18.981	19.477	19.706	528609	8166849	17.18
11	20.003	20.455	20.615	1541758	22314208	46.94
12	20.615	20.688	20.917	225899	2428669	5.11
13	20.917	21.131	21.311	131212	1539631	3.24
14	21.414	21.661	21.866	32962	348373	0.73
15	23.304	23.450	23.518	30556	213298	0.45
16	23.518	23.932	23.985	376593	5092939	10.71
17	23.985	24.082	24.501	578223	6502933	13.68
18	32.636	33.006	33.512	3208685	47534730	100.00
19	36.693	36.780	36.914	137666	826257	1.74
20	37.151	37.305	37.558	1098412	10393044	21.86
21	38.551	38.681	38.868	2156908	16606850	34.94
22	39.615	39.663	39.697	52816	167005	0.35
23	40.179	40.228	40.281	83483	282270	0.59
24	41.200	41.278	41.336	344114	1704043	3.58
25	42.411	42.464	42.528	258032	980014	2.06
26	42.635	42.688	42.742	280048	1049666	2.21

Figure S1. HPLC-DAD chromatogram of Ruta graveolens extract (Mobile Phase: A: 0.1% FA in H2O; B: 0.1% FA in ACN; Injection: 20 µL; Oven 30 °C; DAD: 410, 300 and 250 nm; Column: ZORBAX ECLIPSE XDB-C18, 4.6*150 mm, 5-micron).

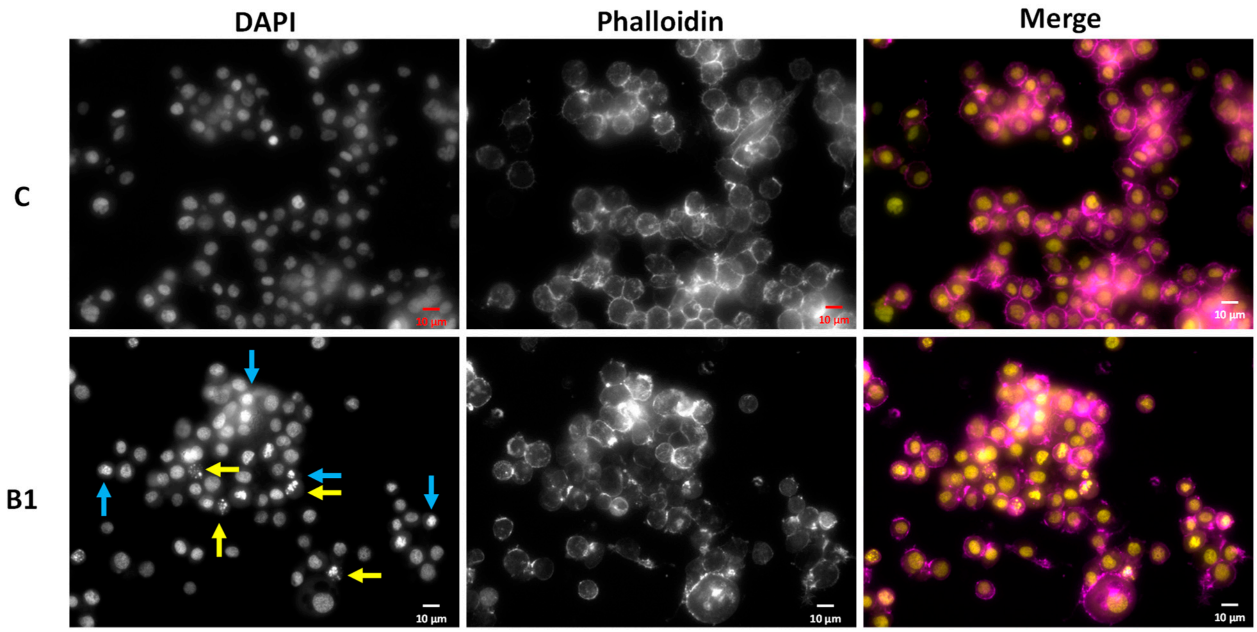

Figure S2. *Sf9* cells exposed to B1 (100 µg/mL) for 24 hours (S Plan Fluor ELWD 40x DIC N1 objective). Cell morphology was evaluated using DAPI (chromatin) and phalloidin (actin). Yellow arrow: chromatin fragmentation; Blue arrow: chromatin condensation. C: Control.

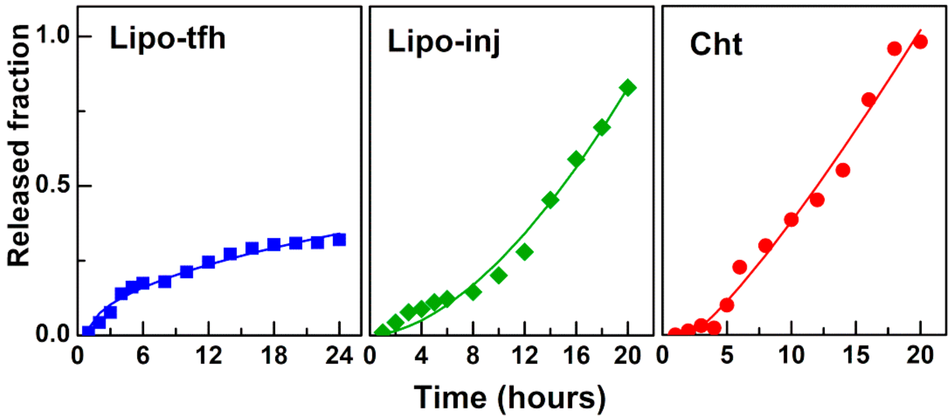

Figure S3. Fittings to Korsmeyer-Pepper model. Lipo-thf: Liposomes (thin film hydration); Lipo-inj: Liposomes (ethanolic injection); Cht: Chitosan nanostructures.

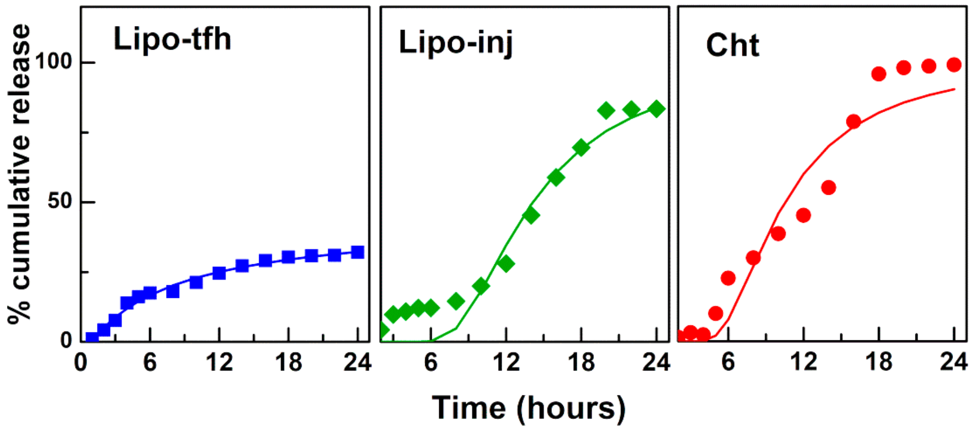

Figure S4. Fittings to Gompertz model. Lipo-thf: Liposomes (thin film hydration); Lipo-inj: Liposomes (ethanolic injection); Cht: Chitosan nanostructures.

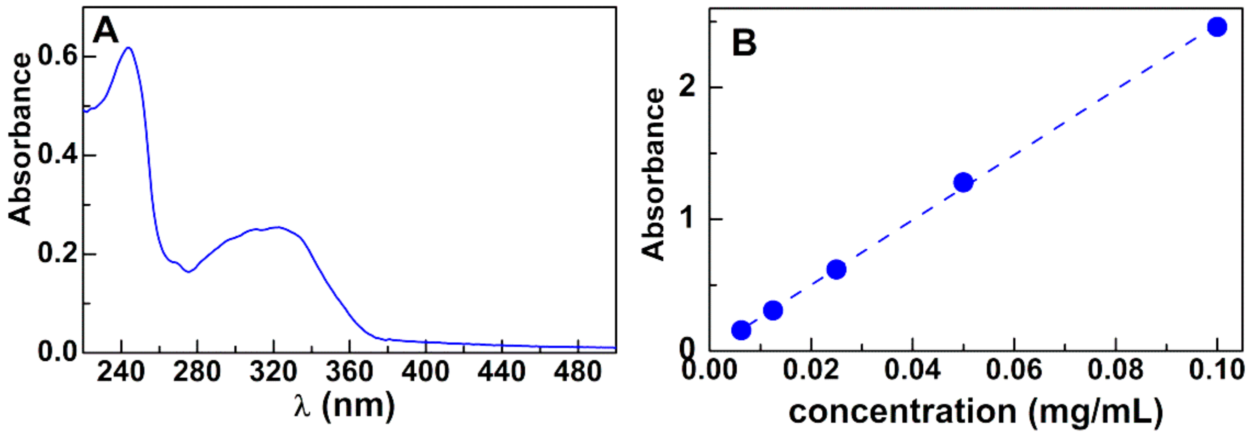

Figure S5. A. UV-Visible absorption spectrum of the extract of *Ruta graveolens* L. B. Calibration curve (absorbance vs. concentration) for determination of encapsulation efficiencies.
